# Food and Trauma: Anthropologies of Memory and Postmemory

**DOI:** 10.1007/s11013-022-09785-2

**Published:** 2022-04-04

**Authors:** Mattias Strand

**Affiliations:** 1 Transcultural Centre, Stockholm Health Care Services, Region Stockholm, Solnavägen 4, 113 65 Stockholm, Sweden; 2 Centre for Psychiatry Research, Department of Clinical Neuroscience, Karolinska Institutet & Stockholm Health Care Services, Region Stockholm, 171 77 Stockholm, Sweden

**Keywords:** Food, Trauma, Memory, Postmemory, Eating disorders

## Abstract

Much has been written about the multifaceted significance of food and eating from an anthropological perspective; the same can be said about the role of food in collective identity construction and nation building. In contrast, the nexus of food, memory, psychological trauma, and disordered eating has been less explored. The aim of this interdisciplinary article is to synthesize available knowledge on this topic by engaging with research literature in fields such as food history, anthropology, sociology, and psychiatry as well as autobiographical works, cookbooks, etc. One main section of the article focuses on the role of food and cooking in exile and refuge. Another section deals with the role of food in the aftermath of historical trauma, whereas a final section discusses various works on disordered eating in the wake of traumatic experiences. In sum, the dual nature of food and cooking—at once concrete and abstract, material and symbolic—offers an arena in which ambivalent memories of trauma can take on tangible form. The concept of postmemory may be useful in understanding how food and cooking can function both as a vehicle and as a remedy for intergenerational trauma.

## Introduction: Food in Memory and Trauma Work

In a passage that has become one of the most well recognized in twentieth-century literature, the narrator in Marcel Proust’s *In Search of Lost Time* is suddenly overcome by childhood memories of Sunday mornings at his aunt Léonie’s house, brought to life by the sensory experience of petites madeleines dipped in lime blossom tea:

And soon, mechanically, dispirited after a dreary day with the prospect of a depressing morrow, I raised to my lips a spoonful of the tea in which I had soaked a morsel of the cake. No sooner had the warm liquid mixed with the crumbs touched my palate than a shudder ran through me and I stopped, intent upon the extraordinary thing that was happening to me. An exquisite pleasure had invaded my senses, something isolated, detached, with no suggestion of its origin. (Proust 1989, p. 48)

At first, the narrator is overwhelmed by the fleeting image and cannot put his finger on it. Little by little, over several pages and not without resistance—“I feel something start within me, something that leaves its resting-place and attempts to rise, something that has been embedded like an anchor at a great depth; I do not know yet what it is, but I can feel it mounting slowly” (p. 49)—the memory fragment reaches a conscious level. Merely seeing the shell-shaped madeleine cake had not been enough to evoke memories of childhood; countless times, the narrator has come across similar cakes on display in the windows of pastry shops without thinking anything of it. Only through the volatile experience of taste is a bridge to the past created.

This episode of the madeleine has often been used to illustrate the newfound technique in modernist literature of turning an external, concrete, and seemingly random incident or sensation into a vehicle that allows the author to change the direction of the story and introduce events that happened long ago in distant places (Auerbach 1953). The fact that a small cake readily takes on this dramaturgical function points to the central role of food and taste in remembering the past. Memories of family meals, recipes passed down through the generations, the tastes of childhood—these are all vital elements that shape our identities and link us to the past, not least in situations where the people, cultures, and societies we grew up in may be forever lost. Through cooking and eating, we navigate and negotiate the family and social relations that shape our biographical selves. Thus, food memories are not solely concerned with tastes and aromas but with people—those with whom we shared dinner, those who cared enough for us to cook us a meal—and with hospitality, trust, reciprocity, and emotional intimacy. Author Wally Lamb describes how his mother’s insistence on always heating up leftovers for him whenever he visited continues to affect their relationship after her death:

Sometimes when I drive to Norwich to visit her grave, I stop by first at D’Elia’s bakery for a Genoa salami grinder. At the graveyard, I weed around Ma’s stone, blow away the dirt that’s settled in the engraved letters of her name. Then I sit beside her stone and devour my sandwich so that she can see I’m still eating. (Lamb 2002, p. 333)

Memories of food are, however, not only associated with nostalgia for the dishes we enjoyed growing up—they may just as well remind us of the jello salads, instant mashed potatoes, or tinned macaroni of decades past that are nowadays often regarded as bland and outmoded (McKenzie and Watts 2020). Of course, this privilege to frown upon certain foods or treat them as amusingly ‘retro’ is intimately tied to socioeconomic conditions (Wills et al. 2008). Moreover, it has been argued that bad cooking may in fact be more memorable than good cooking (Holtzman 2010). Likewise, the food that we as children disliked but were forced or demanded to eat by parents or teachers can remain with us as life-long aversive embodied memories (Batsell et al. 2002). Taste sensations can also be strongly related to memories of poverty and destitution. Many years later, Erik Gideonsson, who was 9 years old at the time, told the story of how his settler family in Swedish Lapland survived the Swedish famine of 1867–1869. After having slaughtered their only cow, eaten the meat, and finally resorted to consuming thin slices of pan fried cowhide, no food remained:

In late winter, we were without food. Now there was nothing but a pair of old boots, but they were tarred and we could not fry them like the cowhide. So we soaked them and boiled them until most of the tar was gone, and then we ate them. But we could not fully get rid of the taste of tar. I can still remember how my throat burned after we had eaten the shoes. (Häger and Villius 1977; translated by the author)

As illustrated above, the flavors of the food we ingest as well as the everyday praxis of cooking and eating play a vital and powerful role in memory work. Much has been written about the multifaceted significance of food and eating from an anthropological and sociological perspective; a brief overview of this field is provided in the section “Methodology and Theoretical Framework” below. As will also become clear, the role of food in collective identity construction and nation building is well documented. In contrast, the nexus of food, memory, psychological trauma, and disordered eating has been less explored. This article is an attempt to address this gap by bringing anthropological literature on food and trauma as related to identity, memory, and postmemory into dialogue with psychiatric research on posttraumatic disordered eating. The aim is to achieve a triangulation where autobiographical narratives, works on food history, anthropology, and research in the field of eating disorders may collectively inform the understanding of food as memory work and the role of cooking and eating in the wake of historical and personal trauma.

## Methodology and Theoretical Framework

### Sources, Outline, and Reflexivity

This is a theoretical interdisciplinary paper based on a large number of original works of a highly diverse nature. In the attempt to synthesize available knowledge on food and trauma as related to identity, memory, and postmemory, this article engages with research literature in fields such as food history, anthropology, sociology, and psychiatry; autobiographical works and works of fiction; cookbooks; and newspaper feature articles. Occasionally, works in visual art will be used to illustrate important themes. These sources have been identified and selected based on extensive literature searches in databases such as Web of Science, Scopus, Academic Search Complete, AnthroSource, Project MUSE, JSTOR, MEDLINE, and PsycINFO. Furthermore, additional literature has been identified through examining reference lists. However, it should be noted that this is not an attempt at performing a *systematic* review on the topic of the role of cooking and eating in the wake of historical and personal trauma. Although not unheard of in medical anthropology (see, for example, Colvin 2015), the systematic review genre is perhaps not the most suitable for research questions of an explicitly interdisciplinary nature where existent data sources vary significantly in terms of style, approach, and audience. For instance, as noted above, the corporeal sensuality that characterizes much food writing tends to blur the line between scholarly and popular endeavors—one example being the cookbook-memoir hybrids that are typically not indexed in academic databases but that nevertheless provided useful data for this study.

The next three subsections provide a theoretical framework with regards to anthropological perspectives on food and eating in a more general sense, the culinary legacy of nationalism and colonialism, and the role of food in memory work. The first main section of this article, titled “The Loss of Culinary Worlds,” focuses on the role of food and cooking in exile and refuge. A number of works on the significance of food among Holocaust survivors are also summarized. A subsequent section, titled “Food and Cooking as Postmemorial Work,” deals with the question of how food and cooking can function both as a vehicle and a remedy for intergenerational trauma. Here, the concept of *postmemory*, as developed by theorist Marianne Hirsch, will be instrumental in discussing the ways in which food and cooking becomes meaningful in the aftermath of historical and personal trauma. Finally, a section titled “Food as Concretized Trauma” focuses more specifically on cooking and eating as an arena in which memories of trauma can take on concrete form. Epidemiological findings on trauma and eating disorders have been summarized elsewhere (Trottier and MacDonald 2017; Molendijk et al. 2017; Caslini et al. 2016); importantly, the emphasis here is on the (few) existent qualitative and ethnographic works on disordered eating in the wake of traumatic experiences.

In what follows, my own exotic horizon as author will occasionally come into play. In particular, a number of examples related to Swedish cuisine—which is substantially less internationally renowned compared to those of many other countries and regions and, to be honest, probably mostly evokes associations to the Swedish Chef on Jim Henson’s *The Muppet Show—*will be used in order to illustrate important points; hopefully, this will not seem overly provincial but instead underscore the universal nature of the relationship between food, identity, and memory. To some extent, this perspective may also counter the clear United States-centric tendency in the scholarly literature on food and identity, reflecting the great interest in the culinary traditions of various immigrant groups to the United States.

### Anthropologies of Food and Eating

Much scholarly work on food, cooking, and eating in anthropology and the social sciences echoes philosopher Michel de Certeau’s suggestion that mundane everyday life practices contain complex and subtle elements of resistance that operate in the fissures of prevailing topographies of power (de Certeau 1984). From this perspective, studying the foodways of various groups enables us to grasp how power relations, desires, and memories become embodied and incorporated. Importantly, this type of embodiment must not be seen as isolated or private phenomena but as shaped by our everyday interactions with others (Weiss 1999). The intimate relation between food and human sensory experience—taste, smell, mouthfeel, interoception, and so on—as well as that between food and *taste* in a broader sociological sense provides a rich ground for ethnographic exploration (Sutton 2010). This orientation did not fully emerge until the 1980s and 1990s, however; for many years, structuralist analyses of food and eating, such as anthropologist Mary Douglas classic *Deciphering a Meal* (Douglas 1972), dominated. An oft-referenced example of how the senses come to life in anthropological writing is author Nadia Seremetakis’ meticulous search for the taste memory of a now practically extinct Greek peach; a sensation that surfaces as a shared cultural artifact, a loss that cannot be fully grasped by younger generations, because “nothing tastes as good as the past” (Seremetakis 1994:1). Likewise, anthropologist Paul Stoller’s account of the simultaneously concrete and symbolic significance of a truly disgusting sauce served by a scorned daughter-in-law can be read as a call for the sharpening of ethnographers’ attention to the senses (Stoller 1989).^1^ As described in more detail below, the history of food has also often offered vivid glimpses into the fabrics of historical geopolitics and cultural hybridity. This is, for instance, evident in anthropologist Sidney Mintz’s work on the political economy of sugar and sweetness, highlighting the role of taste and meal practices in shaping global capitalism: “The first sweetened cup of hot tea to be drunk by an English worker was a significant historical event, because it prefigured the transformation of an entire society, a total remaking of its economic and social basis.” (Mintz 1985:214).

There is, of course, a vast sociological and anthropological research literature on food and ethnicity, class, gender, and their various intersections. Likewise, much has been written about cooking in relation to culture, identity, nostalgia, nationalism, colonialism, exoticism, consumption, marketing, and tourism. This article will certainly not provide an exhaustive account of these bodies of literature. In brief, two general stances can be identified in the discussion of food, identity, and ethnicity. On the one hand, many authors have pointed to the commodification of food and cooking as an example of how dominant forces, typically the ‘Global North’ and/or middle-class segments of a population, desire to consume what they perceive as the authentic culture of the Other in a fetishizing and *cannibalistic* manner. Central to this view is author and activist bell hooks’ description of consuming cultural difference as “eating the other” (hooks 1992), where ethnicity is turned into yet another spice in the kitchen cabinet of the enlightened middle class. Striving for cutting-edge connoisseurship of ethnic food becomes a means of affirming one’s own authenticity for the class dubbed by food theorist Lisa Heldke as ‘food adventurers’ (Heldke 2003). Notably, the outsider position of these food adventurers vis-a-vis the cuisines they explore may even be rendered as an advantage, as if they somehow magically realize the genuine potential of an exotic but static ethnic kitchen: “The natives don’t always recognize their own gems in the rough, and even when they do, they often don’t know how to cut and polish them. They just don’t have the (international culinary) skills.” (Heldke 2003:100) As with all trends, unfortunately, the fads of the food adventurer imply a ‘before,’ when the cuisine in question was not yet desirable, and an ‘after’ when it will no longer be fashionable. Food writer Ruth Tam has described this provocative unreliability:

My childhood home in suburban Chicago always smelled like whatever we were cooking. Visiting us meant cloaking yourself in the scent of haam daan ju yoke beng, a dish of steamed pork and salted egg, or the perfume of mapodoufu, tofu and minced pork with a spicy chili and fermented black bean sauce. I didn’t mind the smells growing up because I wasn’t aware of them. That is, until a high school friend declared my house smelled of “Chinese grossness.” […] But something has changed. In cities big and small, Asian dishes and flavors have become popular among foodies at chic eateries. Foods that were once considered too strong, too spicy, too smelly or too obviously-from-an-animal for my white friends are now on Restaurant Week menus nationwide. (Tam 2015)

From this perspective, a shallow interest in the exotic elements of ethnic cuisine as a resource for one’s own self-realization becomes a prime example of *cultural appropriation*. In anthropological research, analyses of cookbooks are often used to illustrate this tendency. For example, English-language cookbooks devoted to Thai food are typically ripe with stereotypical tourist fantasies of vibrant outdoor food markets, elegant teak furnishings, and subservient women with blossoms in their hair inviting the reader to dinner (Mills 2019; Heldke 2003). Alternatively, a cosmopolitan fondness for ‘melting pot’ tropes and hybrid otherness in whatever form may result in phenomena such as pan-Asian restaurants “that capitalize on the presumed interchangeability and indistinguishability of Asian cultures (i.e., sushi offerings in Chinese buffets or noodle houses that serve Filipino pancit, Chinese chow fun, and Japanese soba)” (Bergquist 2006:143).

On the other hand, experimenting, hybridity, syncretism, fusion, and interchange are typically viewed as the very core driving forces in the history of food and cooking. Needless to say, many (if not most) ingredients and techniques that are nowadays closely associated with a certain cuisine were originally imported from somewhere else, learned, and further developed. Not least, the interchange between the Western and Eastern hemispheres following Christopher Columbus’ arrival in the Carribean in 1492 resulted in the spread of a multitude of edible plants and animals from the Americas to the rest of the world—for example, the chili pepper that is a vital ingredient in a vast number of regional cuisines in South and South East Asia, North Africa, South and Central Europe, etc. originated in what is now Mexico (Kraft et al. 2014). Likewise, by an exchange moving in the opposite direction, the cumin commonly used in Tex-Mex cooking was introduced by immigrants from the Canary Island to Texas (Steinhauer 2014). Migration and diaspora communities continue to influence the way we cook and eat. A case in point is fish and chips, the iconically British dish that was, it seems, ‘assembled’ in the nineteenth century through the contributions of Italian, Irish, and Sephardic Jew immigrants to England and Scotland (McCormack 2019). Nostalgia for one’s country of birth motivates many migrants to try to recreate the dishes they grew up with (Ore 2018), albeit with the ingredients available in the new home country—not seldom, this gives rise to novel hybrid cuisines, such as Italian-American or Nikkei food (Cinotto 2013; Takenaka 2017). Complex international trade and migration routes contribute in shaping local culinary traditions in ways that may at first glance seem highly unpredictable and haphazard; consider, for example, the role of West Indian molasses in the food history of Atlantic Canada (Tye 2008) or the emergence of halal 1950s-themed retro hamburger joints in Lebanese-American sections of suburban Detroit (Kinder 2016). The fact that the food industry and restaurant trade are often relatively accessible sources of economic income for immigrants adds to this pattern:

Arab food, for instance—involving seeds from Lebanon grown in Mexico, trucked to [Detroit], cooked in Arab-Muslim-owned restaurants and served to a variety of customers—was not simply a ‘there’ inserted into the landscape of ‘here’; instead, it was the product of complex commodity chains involving people of many national, ethnic and religious backgrounds. (Kinder 2016, p. 900)

Of course, this type of cultural and economic exchange does not always occur on equal terms or out of free choice; choosing to cook with beans based on pride of one’s heritage or out of culinary curiosity carries a different meaning than having to resort to using them because they are more inexpensive than meat, for example (Long 2004). Even so, cooking will most certainly remain a hybrid culture activity whether or not one likes to call it appropriation. From this perspective, the notion of ‘authentic’ food and cooking seems misconceived, not only because it hinges on cosmopolitan middle-class fantasies of otherness but because there simply is no such thing as an essentialist kitchen.

### National Cuisines and Colonial Bricolage

Theorist Jonathan Metzger emphasizes how both the notion of ‘our own food’ and that of ‘the food of others’ are integral elements of nation building (Metzger 2005). Metzger shows how what is nowadays regarded as traditional Swedish cuisine is a largely modern creation that was more or less deliberately put together in mid-twentieth century in a conscious attempt to establish Sweden as a food nation. Similar tendencies have been observed in many other contexts—in fact, scratch the surface of mostly any national cuisine and a history of imagined authenticity will appear. In Japan, for example, anthropologist Emiko Ohnuki-Tierney has described how rice gradually came to function as a metaphor for a collective self, even though this grain used to be reserved for royalty and nobility while common people relied on other staple foods (Ohnuki-Tierney 1993). The work of anthropologist Richard Wilk depicts how the idea of a distinct Belizean cuisine emerged in the 1960s at the brink of national independence to reflect values of self-sufficiency, in contrast to a typical colonial diet of imported flour and salt meats (Wilk 1999). Likewise, the history of ‘Indian food,’ as outlined by anthropologist Arjun Appadurai, involves the hegemonic standardization of regional Mughlai cuisine—a royal cuisine with its roots in Turko-Afghan and North Indian culinary traditions—at the expense of food typical for other regions of the Indian subcontinent by a growing middle class invested in creating a national identity (Appadurai 1988).

In other contexts, colonialism and postcolonialism provide the backdrop for hybrid and creole foodways. It has been suggested that it is “a harsh but true culinary fact that nothing is so stimulating to the art of cooking as a good long foreign occupation.” (Willard 2001:61) This is, of course, an eloquent simplification. Heldke writes:

Vietnamese cuisine, for example, adopted, with great success, many elements of French cuisine during the French occupation of Vietnam. Is Vietnamese cuisine then “better off” because of the French occupation? This is hardly the lesson I would draw from the example. Rather, I would suggest that it points to the possibilities for creative and flexible resistance to oppression that are present even in a culture’s cuisine. (Heldke 2003, p. xx)

In the context of British imperialism, Ghanaian foodways can be used to illustrate a common colonial intersection of ethnicity and class. In Ghana (as well as elsewhere throughout the Empire), British colonialist rulers brought their own food culture with them overseas—at least those parts that could readily be ‘transplanted’ to the new environment. Local Ghanaian elites that wished to emulate the foodways of the ruling class then incorporated elements of British food into their own cooking, which, in turn, became a benchmark for the common people (Tuomainen 2009). As Heldke points out, colonial history is ripe with examples of creative appropriation of the food of the rulers; “of using foodstuffs forced upon one’s culture in utterly new and unexpected ways, or of preparing the colonizer’s most familiar dishes in ways they’d never before experienced them” (Heldke 2003:166).

Not all colonial interactions followed this pattern. For example, the few British food items that had a lasting influence on local Indian elites include toast with jam, alcoholic beverages, and *curry powder* (Ray 2004). The history of curry powder, which has been told in various versions (see for example Heldke 2003), typically involves active efforts by the British colonial army in the creation of a generic ‘allround’ spice mix that could easily be used in army kitchens and that would connote exotic Indianness to the British at home. Many Indian-style dishes have subsequently come to be regarded as canonically British, some of which—e.g., tikka masala and balti dishes—may actually have been ‘invented’ in the United Kingdom rather than in the Indian subcontinent (Buettner 2012; Highmore 2009). Similar to the Asian-British cuisine, the Chinatown kitchens across the United States saw the birth of distinctly Chinese-American dishes, such as chop suey, chow mein, and duck sauce—not to mention the fortune cookie (Davis 2002; Miller 2006). Chinese hybrid cuisines exist in many other places, of course. Much like in the United States, Chinese-Canadian food has become ubiquitous: “A typical vision of a rural town in Alberta is that it has a grain elevator, a hockey rink and a Chinese restaurant (plus the Chinese family that runs it)” (Smart 2003:318).

### The Role of Food in Memory Work

It is beyond the scope of this article to provide an exhaustive account of the philosophical, historiographic, and neuroscientific literature on the role of memory in the life of individuals as well as in society. A landmark work on the phenomenology of memory and the parallel acts of remembering and forgetting is philosopher Paul Ricœur’s *Memory, History, Forgetting* (Ricœur 2004). Here, Ricœur explores the fundamental questions of how an absent past can come to life in the present as memories, how our concepts of historical truth are shaped by the elusive nature of memory, and how it is inevitably necessary to forget in order to remember. Interestingly, in a similar examination of the reciprocal relationship between remembering and forgetting, anthropologist Paul Connerton highlights the history of cooking as a prime example of this duality (Connerton 2008). Connerton notes how the standardization and commodification of modern cuisine largely hinges on the production of printed cookbooks, whereas, in contrast, local practices of food and cooking tend to be “tied to what grandmother did” (p. 64) and passed on in the form of tacit knowledge rather than as recorded recipes. As will become clear from the exposition below, these two forms of food memory often interact in shaping the way we deal with identity and trauma.

An obvious point of reference for anyone interested in food and memory is the work of anthropologist Jon Holtzman (2006, 2009). In an excellent review, Holtzman outlines the various strands of research on food as a means of remembering or making sense of the past: e.g., food as a central element of ethnic identity, nationalism, and invented communities; food as memory-making in exile; food as nostalgia; food as sensous memory; food as gendered narrative; or food as a marker of social transformation (Holtzman 2006). In Holtzman’s account, however, it appears as though even the most nuanced of the referenced works fall short of elucidating the multifaceted relationships between food and memory. In fact, after reading Holtzman, one might begin to doubt whether it is at all possible to add anything of substance to this meandering literature. Holtzman describes the field as underdeveloped—yet, this is not caused by a lack of scholarly attempts but rather due to the large heterogeneity and ambiguity of the phenomena under study: “Each half of this relationship—food and memory—is something of a floating signifier” (Holtzman 2006:362) that scholars typically fail to define adequately. In discussions of memory, it is often unclear exactly which mnemonic faculties and processes (historiography and notions of historical ‘truths,’ memorialization as a political tool, cultural heritage and collective memory, nostalgia and loss, embodied or ‘practical’ memory, neurobiological networks mediating memory on conscious and/or unconscious levels, etc.) that are the focus of attention. Likewise, food and eating may be viewed as biological necessities, as inherently social arenas, as a sensual experience, as signs of reciprocity, as economic transactions, as cultural constructs, and so on. Entering this maze, one is bound to get lost among the various intersections—or is the real danger staying on the beaten path, repeating the same old tropes? For example, is it not rather unimaginative to once again invoke Proust’s madeleines as an introductory illustration for a text on memory? Can any attempt to deal with food and memory ‘succeed,’ given the elusive nature of both entities?

Certainly, there are many important points to take away from reading Holtzman. Importantly, he points to a tendency in much of anthropology to treat food and taste as stereotypically ‘Western’ forms of sensuality, drawing on epicurean sensibilities that are deeply situated in and particular to a dominant ideological and phenomenological discourse whereas hunger, lack, discomfort, and sickness may actually be more relevant points of reference in many parts of the world (Holtzman 2006, 2010). This corporeal sensuality—“the catnip so many of us enjoy in food studies scholarship” (Gálvez 2020:545)—also endow scholarly literature on food with an unusual allure: “That is, where a book on structural adjustment programs, for instance, has little potential for popular appeal, a book on camembert has potential marketability among high-brow, deep-pocketed cheese lovers.” (Holtzman 2006:364) Although many phenomena exist on a spectrum of materiality and abstraction, few are at once as deeply sensuous and symbolically charged as food. Interestingly, Holtzman suggests that this very nature of food makes it a suitable vantage point from which to approach the equally ambivalent and layered concept of memory in order to arrive at a synergistic whole, “albeit a messy and ambiguous one” (p. 374).

## The Loss of Culinary Worlds

The very same elements that render food and cooking important in constructing national identities can also be turned around and put to use in the destruction of communities. In militarized conflict, food—or, not least, the withholding of food—can become highly politicized and exploited as a weapon (Collinson and Macbeth 2014). The impact of food insecurity in areas of conflict and postconflict is well documented; less has been said about the relationship between conflict, food insecurity, and everyday cooking culture and heritage. On a fundamental level, the eradication of culinary traditions has been attempted for purposes of supposed assimilation or even genocide (Mintz 2019; Daschuk 2019; Nilsson et al. 2011; Green 2016). During the Khmer Rouge regime in 1970s Cambodia, targeted food rationing and altered agricultural policies resulted in mass starvation, contributing to the death toll of two million Cambodians between 1975 and 1979 (Tyner and Rice 2015). Moreover, records of traditional Cambodian cuisine were destroyed by the Khmer Rouge as part of their increasingly paranoid efforts to cleanse the nation of anything bourgeois—indeed, being found cooking could result in a death sentence (McCafferty and Tham 2017; Taylor 2018). After the fall of the Khmer Rouge, the Cambodian culinary heritage was forgotten, sidelined, or lost (Taylor 2018) and only through dedicated efforts have the country’s traditional dishes slowly started to make a comeback. The impact of militarized conflict on the foodways of ordinary people often leaves tangible traces in the landscape. In the Central African Republic, for example, mangoes decomposing on the ground characterizes the sites where Muslim Peul families used to live before they were forced to flee by Christian militias:

You can always tell where there used to be a village by the sudden profusion of mango trees in the middle of nowhere. […] At this time of the year, most of the fruit is still small, hard and green. It's hard to imagine how, by June, the ground below will be covered in a rotting yellow squelch, the aroma more sickly than sweet. (Whewell 2014)

From a combatant perspective, the dreadful hunger experienced during war and the camaraderie associated with finally having the chance to eat with your peers may become entangled in memory. In postconflict Northern Mozambique, ex-militia soldiers do not primarily recall tastes and smells from their days of hiding out in the forest—the dried and pulverized banana tree roots they survived one did not taste much (Katto 2020). Rather, what they remember is the communal act of eating together in the field, which also melts into idealized childhood memories of sharing food within the family. Still, bitter postwar experiences of impoverished communities, weakened social ties, and an increasingly consumerist relation to food “make a narrative of sweetness impossible” (Katto 2020:983). For these ex-combatants, the hopes of liberation becoming a vehicle of social transformation and justice did not materialize; they starved for nothing.

For refugees, the food of home may take on a special significance. As noted in a study on Sudanese asylum seekers in Tel Aviv, finding the time to relax over a plate of kisra bread and okra may provide some “temporary relief from constant engagement with unfamiliarity” (Sabar and Posner 2013:207). People in forced migration settings often have to share accommodation with others and work long hours, which leaves little room for socializing around food at home. Moreover, in gendered migration contexts where a majority of asylum seekers are men, many may not be used to cook for themselves (Sabar and Posner 2013). Communal meals in quasi-commercial restaurants or bars with people of the same nationality may provide a sensuous connection to the land one has been forced to flee and some continuity in a highly uncertain situation. Similar tendencies have been observed among Dominicans in New York City, for whom food and cooking as well as the very process of acquiring necessary ingredients through navigating local commercial networks of fellow countrymen help connect past and present (Marte 2011). Here too, restaurants run by Dominican immigrants “offer a surrogate domesticity as substitutes for home-cooking and family niches” (p. 186). However, even when refugees have the opportunity to eat dishes from home, many experience a ruptured connection to the soil—to the embodied meanings associated with growing and harvesting that cannot be compensated for (Sabar and Posner 2013). Altered diasporic foodways may also give rise to feelings of shame; for example, among elderly Zainichi Koreans in Japan, a lost ability to consume kimchee and other spicy Korean foods (due, perhaps, to peptic ulcers and the like) has been linked to humiliating experiences of rootlessness in the face of longstanding discrimination (Lee 2000).

In her work, visual artist Lap-See Lam addresses themes revolving around memory, migration, authenticity, and racism from a Chinese-Swedish perspective. Lam’s parents and grandmother opened the restaurant Bamboo Garden in Stockholm in the 1970s and ran it for 35 years. During this period, the mainstream perception of Chinese food in Sweden has changed, from overly exotic to accepted and eventually so commonplace that it has almost become invisible. Here, Chinese restaurants have largely served as a socioeconomic stepping stone for Cantonese, Taiwanese, and Chinese-Vietnamese immigrants arriving in the country in the 1970s (Kjellgren 2001). For many restaurant owners, the business has been a first stop on a road to a better life and their children are expected to pursue careers in other professions rather than take over the restaurant (Curman 2018; see also Smart 2003). Similar to the Chinese-American kitchen, the Cantonese cuisine in Sweden was adapted to available foodstuff and organized according to typical Swedish mealtime customs by which, for example, everyone orders their own main dish instead of sharing multiple dishes. This hybrid Chinese-Swedish kitchen is emblematic for ‘Chinese food’ for many Swedes, but has also increasingly been viewed as old-fashioned, inauthentic, and kitsch. As the original owners grow old, retire, and sell the restaurants, many of these establishments are lost, along with an underdocumented and little researched part of contemporary Swedish cultural history. In Lam’s words, “so much about these places remains untold, because those of us that were brought up in this environment were raised to avoid sentimentality” (Curman 2018, pp. 18–19; translated by the author). As an artistic response, Lam has created three-dimensional scans of entire Chinese restaurant interiors—eerily familiar but lost landscapes for the audience to explore with the help of virtual reality headsets.

In many post-Holocaust works of literature, there is a strong nostalgia for the cultural landscapes of a ‘lost world’ devastated by the two World Wars. The longing for the food of the innocent or peaceful decades past is an integral part of this tendency, which involves Ashkenazi Jewish cooking in particular but also memories of turn-of-the-century Vienna or Belle Époque restaurant and café culture in a more general sense (see for example Zweig 1943). In his memoirs, Hungarian-American restaurateur George Lang recalls the tangerines, roasted chestnuts, and boiled beef and marrow of his childhood 1920s Hungary. Decades later, after his family and the friends he grew up with have been sent to the Nazi gas chambers and he himself has narrowly escaped when Russian forces liberate Budapest, what he remembers most vividly from his early years is a Bach cantata and the aromas and flavors of his mother’s kitchen (Lang 1998). His later work in the lavish world of the New York City food and entertainment business has been described as a way of drowning out the haunting memories of starvation and death with fine dining abundance (Rosofsky 2004). However, childhood memories notwithstanding, most Ashkenazi Jews in pre-war Eastern Europe survived on a diet of a potatoes, herring, dark rye bread, salt, and garlic (Diner 2001)—clearly, the foods of the poor people are not always chosen for remembrance (see also Metzger 2005).

Half a century after the Holocaust, a crumbling copybook finally found its way to the descendants of Mina Pächter, a Jewish woman who starved to death in the Czech Theresienstadt hybrid ghetto and concentration camp. The book turned out to be a haunting document, containing recipes of favorite Bohemian and Moravian dishes collected among women inmates in the face of annihilation: potato salad, cherry-plum dumplings, goose neck stuffed with semolina (de Silva 1996). Some recipes are incomplete, indicating perhaps that the contributor was suddenly deported onwards to an extermination camp. Ingredients that were scarce or unavailable in the ghetto, such as honey or coffee, are sometimes left out or marked as optional. In the recipe for cold stuffed eggs, we are instructed to “let fantasy run free” (p. 52)—indeed, being able to fantasize must have been of utmost necessity for survival in the camp:

Did setting down recipes bring comfort amid chaos and brutality? Did it bring hope for a future in which someone might prepare a meal for them again? We cannot know. But certainly the creation of such a cookbook was an act of psychological resistance, forceful testimony to the power of food to sustain us, not just psychologically but spiritually. (de Silva 1996, p. xxvi)

Witnesses such as Primo Levi and Viktor Frankl have stressed how daily life in the Nazi concentration camps—the bare surviving in the face of utmost despair and hopelessness—revolved around *bread* (Levi 1959; Frankl 1959). Prisoners dreamed not of extravagant foods but of bread; in the informal camp economy, bread was a main currency. This utmost importance of bread is captured in ethnographic work on traumatic embodiment among regular visitors at a support facility for aging Jewish Holocaust survivors in London (Kasstan 2015). Here, the everyday menu is “a conscious strategy of care” (p. 349) where, for example, a wholesome bowl of Polish barley soup may represent childhood memories as well as the recipe for survival. At this facility, importantly, bread is never allowed to go to waste—in the words of one of the survivors, “I waste a lot of things in the kitchen, but when it comes to bread I do anything with it but I can’t throw it away because people were dying for a *crust* of bread” (p. 356; italics in original). Just outside the kitchen, there is always a plate of traditional rye bread with caraway seeds that visitors can help themselves to: “It is not uncommon to see the slices wrapped into a handkerchief and slipped into a handbag, or to find rolls of bread bulging out of blazer pockets ‘just in case’.” (p. 355) Bread equals safety; the embodied memory of decades-old survival strategies whereby a tucked-away slice of bread could be the difference between life and death. Interestingly, a tendency for hoarding food has been described among Holocaust survivors as well as among prison camp survivors in general. Holocaust survivors residing in the United States have described how in the concentration camps, out of desperation, they had to resort to eating grass, leaves, or paper and how these experiences have shaped their later attitudes to food and eating. For example, throwing away food (even when spoiled) is seen as a sin by many, excessive amounts of food is kept at hand, and a majority report feeling uneasy when standing in line at a restaurant due to camp memories (Sindler, Wellman, and Stier 2004).

## Food and Cooking as Postmemorial Work

A useful concept in the discussion of food and trauma is that of *postmemory*, developed by theorist Marianne Hirsch and others. For Hirsch, postmemory refers to the particular impact that those traumatic memories that are handed down—explicitly or, perhaps more often, implicitly—have on the children and grandchildren of trauma survivors:

“Postmemory” describes the relationship that the “generation after” bears to the personal, collective, and cultural trauma of those who came before—to experiences they “remember” only by means of the stories, images, and behaviors among which they grew up. But these experiences were transmitted to them so deeply and affectively as to *seem* to constitute memories in their own right. Postmemory’s connection to the past is thus actually mediated not by recall but by imaginative investment, projection, and creation. (Hirsch 2012, p. 5; italics in original)

Importantly, while postmemory is not the same thing as a memory of an event experienced at first hand, “it approximates memory in its affective force and its psychic effect” (Hirsch 2012:31). In order to illustrate the ‘post’ nature of postmemory, Hirsch evokes the image of a post-it note: a message that adheres to something else (i.e., a lived trauma) but that might just as well fall off and acquire an independent artefactual quality. In this way, postmemory can resemble a palimpsest; a surface on which the original text has been partly erased and later writing superimposed. Naturally, one might argue that memory in general also behaves in this way, considering the active and creative input (on a neural as well as on a social and relational level) of the individual in shaping and attaching meaning to what becomes memories through directed attention, processing, forgetting, and continuous re-evaluation. Likewise, the significant role of *repetition* that have been said to characterize the postmemorial generation in their efforts to grapple with inherited trauma (Hong 2020) is, of course, evident in traumatic memory in general in the form of intrusive flashbacks, reoccurring nightmares, etc. What distinguishes postmemory as a transgenerational phenomenon is the tendency of the prior layers of the palimpsest—the traumatic memories of events experienced by others—to surface in fragments that bleed through one’s current life story and become visible at unexpected moments. Hirsch stresses that “postmemory is *not* an *identity* position but a *generational* structure of transmission” (Hirsch 2012:35; italics in original)—from this perspective, primary importance is given to the modes of representation within a family and in society at large, rather than to any ideas of inherent characteristics associated with survivorship.

Hirsch’s account of postmemory focuses on the experiences of the children and grandchildren of Holocaust survivors. Research on the first-generation survivors themselves has displayed a remarkable resilience from a psychiatric perspective; although many have suffered from subsequent depression and/or posttraumatic stress disorder (PTSD), the symptoms have often become attenuated over the years so that about half of them achieve partial or full recovery (Trappler, Cohen, and Tulloo 2007, 2002). As outlined above, altered eating habits related to previous starvation have been described among Holocaust survivors, although some studies have not found any long-lasting consequences in terms of eating pathology (Bachar, Canetti, and Berry 2005). Similarly, among the second and third generations, disordered eating is not predicted by the level of the individuals own postmemorial exposure to Holocaust stories and imagery. Interestingly, however, second-generation mothers who have experienced more Holocaust exposure have daughters who, as a group, display more eating pathology (Zohar, Giladi, and Givati 2007). The authors suggest that this pattern of “generation skipping” may be due to a tendency among survivors to keep their Holocaust memories to themselves and not discuss them with their children in order to achieve a semblance of normality; however, “[a]s the Holocaust survivors aged, there was a tendency for them to give testimony, to share with their grandchildren memories that they had avoided thinking and talking about” (Zohar et al. 2007:55). Still, arguably, this type of narrative exposure is more readily integrated into a broader societal context, whereas the postmemorial silence experienced by the second generation—the *phantom* famously depicted by psychoanalysts Mária Török and Nicholas Abraham (1994)—becomes more intimately woven into everyday life.

Yet another postmemorial phantom haunts the kitchens of southern United States and the Caribbean: that of *slave food*. The nostalgia and romanticism associated with the notion of the South and ‘southern food’ is well documented (see for example Wallach 2016). Regardless of whether the era in question is that of antebellum slavery or postbellum plantation culture and sharecropping, the food that black slaves and ex-slaves cooked and ate is at the very core of southern cuisine. It can be argued that food spaces throughout the Jim Crow South were used both to draw the lines of racial segregation and, later, to dismantle the very same system—for example, lunch counter sit-ins became a central element of 1950s and 1960s anti-discrimination activism (Cooley 2015). Nevertheless, the history of the Atlantic chattel slavery is, of course, still ever-present in the United States (and elsewhere). Literary scholar Christina Sharpe evokes the postmemory of contemporary blackness in her book *In the Wake: On Blackness and Being*. Here, ‘the wake’ takes on a multitude of fateful connotations, such as the track left on the surface of the sea by a ship, the state of wakefulness, the recoil of a gun, the watch held beside the body of someone who has died, or the ritual mourning and celebration of the life of the buried (Sharpe 2016). The same uncanny ambivalence can be found in relation to the chitterlings, pig’s feet, neckbones, and rice middlins of slave cuisine: this is, at least by some accounts, ‘comfort food,’ designed to fill the belly of hard-working agricultural laborers. It is also, however, a cuisine based on the scraps and leftovers that the masters did not care for.

Since few original recipes from the era of slavery have been documented, cooks who wish to prepare the dishes of slaves in order to honor a cultural heritage often have to make do with their later reincarnation in the form of ‘soul food’ (Halloran 2012). This cuisine, strongly influenced by the traditional foodways of West Africans and Native Americans, can certainly be a great source of cultural identity and pride. A tendency of embracing the culinary heritage of slaves is evident not only in the ‘Old South’. For example, mangú—i.e., boiled plantains mashed with butter or oil—is another “former slave food which has become an index of racial identification and affirmation of ‘blackness’” (Marte 2011:193) among Afro-Dominicans in New York City. Indeed, the plantain as such has become a stereotypical yet iconic symbol of Dominican, Puerto Rican, and Caribbean culture. Artist Miguel Luciano has made use of these associations in his 2006 work *Pure Plantainum* where, for instance, a platinum-plated plantain becomes the main pendant of a massive braggadocio-style Cuban link neck chain (Mosaka et al. 2007).

Criticism has, however, also been voiced against tendencies to transform and beautify the food legacy of slavery. In his “culinary memoir” of childhood in Barbados, author Arthur Clarke asserts: “Slave food doesn’t have a damn thing to do with the soul or with ‘black is beautiful’. It has *everything* to do with the belly.” (Clarke 2014:60; italics in original) Slaves were typically left with the pieces of food that nobody else had the heart to eat or with whatever they could grow in the backyard or forage in the plantation fields at night; ingredients that they then had to cook in a single pot out of necessity since they usually did not own other utensils. In this account, slave food was food to survive on after toiling from sun-up to sun-down, rather than an expression of soulfulness. Analogous to discussions of the nature and significance of Black ‘soul’ in general, soul food as a culinary category has been highly divisive. Whereas proponents such as author Amiri Baraka argued that cultural forms born out of a history of oppression could be a source of pride, the Nation of Islam as well as the Black Panthers tended to dismiss the foods associated with the era of slavery as unclean and unhealthy—the latter described soul food as a fad propelled by a Black middle class ‘gone slumming’ (Witt 2004). Moreover, it has been suggested that many soul food restaurants are more ‘Southern’ than ‘soul’; i.e., that the focus is typically on rich and hearty dishes rather than on the hog bowels and ashcakes that most slaves got by on. This, perhaps, reflects the split nature of slave existence highlighted by culinary historian Michael Twitty, noting how the everyday lives of enslaved plantation cooks shifted between their own cramped cabins and the Big House kitchen (Twitty 2017). Certainly, West African cooking traditions (and ingredients imported on the slave ships) had a major influence on the blue crab and waterfowl dishes of the Chesapeake, the catfish stews, crab cakes, and sweet red rice of the South Carolina Lowcountry, or the Creole jambalays of the Gulf Coast. Even so, this was not the food that the slave cooks themselves ate—their diets were, with “little variation from Maryland to Missouri, from Texas to Mississippi to Florida” (Twitty 2017:214), characterized by hominy and hoecakes, the cornmeal-based foods that contributed to widespread nutritional deficiencies such as pellagra among slaves. Even the stereotypical watermelon was often off limits to the enslaved population. The plantation kitchens in all their culinary richness and the slave quarters, although inhabited by the same cooks, were worlds apart. Big House kitchens were, however, not merely sites of cooking craftmanship. Postmemorial ghosts haunt these places too:

The kitchen is where we acquired the eyes of our oppressors, their blood and bones and cheek-blush. The kitchen was, perhaps more than any other space during slavery, the site of rape after rape, sexual violations that led to one of the more unique aspects of African American identity—our almost inextricable blood connection to white Southerners. (Twitty 2017, p. 107)

A particular example of postmemorial work is that of international adoptees, i.e., individuals who are adopted, typically at an early age, to a country other than that in which they were born and who have often been provided with little or no information about their birth parents. International adoptees may come to inhabit an ambivalent position in relation to their countries of birth as well as to their adoptive countries: being at once inside and outside, having had little access to cultural knowledge, identity, and pride that others, based on racial stereotypes, presume that they possess (Wiley 2017; Palacios et al. 2019). As outlined above, food has become a marker of ethnic group membership and sought-after authenticity; hence, for adoptees, the relationship to the cuisine of one’s birth country as an access point to personal identity may be highly ambiguous. Among adoptees born in South Korea, for example, it has been noted that embracing Korean food can serve as an important way of connecting with other adoptees as well as “a kind of Diasporic Return to Korea, birth families, and cultural heritage” (Riel Müller 2016). Here, pre-adoption non-episodic memories of smell and taste—real, imagined, or perhaps a bit of both—may contribute to the experience of affective connection and continuity (Bergquist 2006). However, appreciating the cuisine of one’s birth country may also turn into a requirement or a yardstick for measuring attachment. What if, as a Korean adoptee, one is not particularly fond of Korean food? It has been observed that some adoptive parents tend to view their children’s preference for well-known meatballs, rød pølse, or hamburgers over the largely unfamiliar foods of their countries of origin as a sign of them ‘really’ being more Swedish, Danish, or American than Korean, Indian, or Ethiopian (Bergquist 2006). Of course, since many children naturally display a significant novelty aversion when it comes to food, such assumptions easily become self-fulfilling prophecies. Moreover, supporting adoptee children in exploring their birth heritage ideally amounts to more than taking them to ethnic restaurants or teaching them how to eat with chopsticks: “While understanding the symbolic, metaphoric, and affective meaning that kimchee and moon cakes represent for [Korean] adoptees, it is equally important to note that their consumption is not always necessary and in isolation, insufficient. Adoptees will be Chinese American or Korean American whether or not they ever eat dim sum or bulgogi.” (Bergquist 2006:150) Food is certainly a marker of collective heritage; it is, however, also a means by which one may set oneself apart (Riel Müller 2016) or even become excluded from communities.

## Food as Concretized Trauma

The inherent hybridity and everyday nature of cooking offers an arena in which ambivalent memories of trauma can take on concrete form. This process, however, may also contribute to normalizing traumatic history, making it opaque for later generations. An example of this phenomenon is budae jjigae, or ‘military base stew,’ which has become a typical dish in South Korea and the Korean-American diaspora. Budae jjigae was created in United States military kitchens during the Korean war and is made from food items that were readily available in this context: a blend of local ingredients such as cabbage, kimchi, and gochujang chili paste and the staple canned goods—ham, hot dogs, baked beans—provided by the United States army (Woodcock 2018). After the war, budae jjigae survived as a simple and filling dish. Today, however, it tends to be viewed as cheap and bland, an awkward outsider in relation to the ‘authentic’ Korean culinary heritage. The somewhat odd mix of kimchi and hot dogs is seen by many elderly Koreans as disgraceful, whereas younger generations look upon budae jjigae as a slightly embarrassing gimmick—the type of unsophisticated and outmoded dish that one might remember from school outings decades ago. However, it has been suggested that this mockery ignores and trivializes the dark history behind the creation of the dish: the fatal war that split a nation, separated families, and paved the way for authoritarian regimes (Woodcock 2018). Much like how the Korean War has been described as a forgotten war, overshadowed by the Second World War and the Vietnam War, the scolded and ridiculed budae jjigae is a culinary heritage that many would prefer to forget.

The concrete-yet-abstract duality of food can also shape the experience of grief after the death of a loved one. In a recently published cookbook, the mothers of Mexican *desaparecidos*—the large number of young people who have disappeared more or less without a trace in the wake of the wars between drug cartels—collect their lost children's favorite recipes in what amounts to a culinary grief work (Gómez Lucini and Las Rastreadoras del Fuerte 2020). Here, the favorite dishes become proof of the very fact that the deceased used to exist and that they are not forgotten. And, once again, it is the uncomplicated cooking that most readily lends itself to representing the memory of the dead: “It’s very simple, but it was my son’s favorite meal […]. He called them ‘pizzadillas’ because I’d use two tortillas, instead of just folding one in half. Then, my son would cut it into four pieces, just like pizza slices.” (Barajas 2020). It is not in the most elaborate dishes but rather in simple everyday food—bread, grits, hot dog stew, a quick tortilla pizza that a son used to grab and eat on the go—that traumatic memory becomes embedded.

In the field of eating disorder research, an overarching focus on eating pathology as a maladaptive coping mechanism in response to affect dysregulation (Trottier and MacDonald 2017) has resulted in food being regarded merely as yet another arena for internalizing behavioral problems (Strand 2021), alongside other self-injurious behaviors, use of illicit substances, etc. With the possible exception of narratives of patients’ lived experiences (see for example Brewerton, Alexander, and Schaefer 2019), there have been few ethnographic or even qualitative inquiries into trauma, food, and eating disorders. One example of research focusing on the concrete aspects of eating and trauma is a qualitative study among United States veterans (Smith, Klosterbuer, and Levine 2009). Here, ex-military personnel were interviewed in depth about the impact of military service and deployment on their eating behaviors. Overall, the veterans described how they developed a highly ambivalent relationship to food during their time in the military. On the one hand, there was typically plenty of food available in the mess halls during basic training and many soldiers living on base ate large amounts due to the physical requirements and high activity levels. On the other hand, food insecurity was a perpetual problem for those involved in combat—often, they could not carry everything they needed and would sometimes have to starve or resort to catching and eating rats, snakes, dogs, or monkeys. Whenever food became available during deployment, soldiers tended to eat as much as they could and to do it as fast as possible. Many also learned to stash food. Notably, this alternating pattern of food abundance and insecurity during military service—perhaps most sharply experienced by those who had been prisoners of war—was associated with post-service changes in eating behaviors. The veterans described how they would continue to hoard and overeat high-calorie foods when they were no longer in the military; habits that typically led to weight gain due to a simultaneous decline in energy expenditure. Many also suffered from PTSD post-service and reported turning to food as an escape from mental pain. Alternatively, certain foods could aggravate symptoms of PTSD—in particular, eating rice would trigger intrusive flashbacks to wartime Vietnam or Korea for some veterans.

For lack of medico-anthropological publications exploring everyday lived experiences of trauma and eating disorders, one might instead turn to works of autobiography and literary fiction. In Edwidge Danticat’s novel *Breath, Eyes, Memory*, published in 1994, we follow Sophie who is sent from an impoverished Haitian village to New York City at the age of twelve—just like the author—to live with her mother who migrated before her and who she barely remembers. In the United States, triggered by the availability of cheap foods, the different expectations in terms of ideal body shape, and the violence witnessed as a child growing up in the Baby Doc era, Sophie develops bulimia nervosa. Her mother details her own experiences of coming from Haiti to the United States:

In the beginning, food was a struggle. To have so much to eat and not to eat it all. It took me a while to get used to the idea that food was going to be there to stay. When I first came, I used to eat the way we ate at home. I ate for tomorrow and the next day and the day after that, in case I had nothing to eat for the next couple of days. I ate reserves. I would wake up and find the food still there and I would still eat ahead anyway. (Danticat 1994, pp. 179–180)

Sophie’s utterly unfamiliar experience of bingeing and purging—“I have never heard of a Haitian woman getting anything like that. Food, it was so rare when we were growing up. We could not waste it.” (p. 179)—is embedded in the confusion arising from being brought up in a hand-to-mouth context of ‘eating ahead’ and then suddenly being challenged by an abundance of highly palatable energy-dense foods. Notably, the scarcity of the poor Haitian village is depicted within a framework of surviving *together* off the land, whereas the New York abundance is oblivious of any such environmental and relational aspects. The Haitian connection between food and soil stands in sharp contrast to the lackluster Brooklyn mother-and-daughter meals, “not cooked but simply heated or boiled quickly” (p. 99), which come to epitomize the transformation of eating from a communal activity to an act of detached consumption. Here, material excess implicates social impoverishment, mirroring an ambiguity in terms of abundance and scarcity—or, if you will, pleasure and trauma—that has arguably become central to dystopian notions of modernity and progress.

## Concluding Discussion

The dual nature of food and cooking—at once concrete and abstract, material and symbolic—is evident throughout the ethnographic and autobiographical accounts referenced above. Historical and personal trauma, filtered through processes of memory and postmemory, shapes our everyday dinner plates. The food we eat reads as a map of centuries-old geopolitics and forgotten injustices as well as of our present cultural and socioeconomic topography. Yet, our foodways are not simply ‘who we are’ in culture—they arise out of the (at times exotifying or racist) expectations of others, out of uneasy communities and compromises. The act of cooking is never either authentic or appropriated; more often than not, what we perceive as genuine rests upon a complex history of exchange and exploitation. In the here and now, food and eating is inevitably tied to our perpetual oscillation between hunger and satiety. Food may nurture our minds and our spirits; when bad, however, it makes us dreary, offended, or even hostile. Not least, of course, it provides the very building blocks of our human bodies.

This article has outlined some of the many ways in which these various aspects of the everyday acts of cooking and eating may perform memory work in the wake of historical and personal trauma. It has become obvious that the dual, material-cum-symbolic nature of food and cooking offers an arena in which ambivalent memories of trauma can take on tangible form. This duality is evident in, for example, the slave food/soul food of southern United States and the Caribbean, the ever-present pieces of rye bread at the support facility for elderly Holocaust survivors, the cringeworthy Korean budae jjigae, or the memories of the everyday favorite snacks of young victims of community violence in Mexico. For refugees and other displaced people in exile, the opportunity to eat the food of one’s home region may entail both a sense of continuity with a lost past and evidence of a ruptured connection to the soil that cannot fully be compensated for. The clash between experiences of severe scarcity and a newfound abundance of food can become a vehicle for commemorating the loss of a culinary culture and drowning out memories of starvation, as in Lang’s work in the lavish restaurant scene of New York City. In contrast, for Danticat’s young Haitian immigrant to the United States or the ex-military personnel used to surviving on what little they can find in the field, the very same clash may ultimately turn out to be very difficult to handle, symbolizing as it does a simultaneous loss of purpose and coherence in everyday life.

Equally ambiguous are the postmemorial accounts of food and cooking. For the children and grandchildren of trauma survivors, an interest in traditional foods can undoubtedly become an approachable way of exploring one’s roots. As folklorist Andriy Nahachewsky has noted, postmigration generations may develop a relation to their parents’ cultural heritage that is at once intimate and independent (Nahachewsky 2002). This may result in highly initiated as well as experimental takes on the cooking of ‘the old country’. Likewise, pride in the recipes born out of the struggles of oppressed ancestors is evident in many communities, not least in the descendants of American slavery or Holocaust survivors discussed in this article. Of course, similar sentiments of continuity and authenticity have been evoked in the creation of many national cuisines across the world, regardless of the factual origins of the dishes chosen for inclusion. However, as described in the case of international adoptees, it is far from certain that individuals who grow up immersed in another food culture than the ones of their birth countries actually *like* the cooking that is associated with their origin—a scenario that may give rise to feelings of dissonance, estrangement, and shame. On the other hand, if food is presented as the *only* readily available connection to the past, which is sometimes the case for international adoptees or other ‘postmemorial’ generations, it may also become a sign of rupture and loss.

The association between trauma, PTSD, and eating disorders is well established in the psychiatric literature (Trottier and MacDonald 2017; Molendijk et al. 2017; Caslini et al. 2016). From an ethnographic point of view, however, one might be surprised by the lack of attention to food as a cultural sphere and the concrete acts of cooking and eating in the research literature on eating disorders—indeed, there seems to be a greater interest in these aspects of food in the fields of experimental psychology, crossmodal sensory perception, and appetite than in the field of eating disorders (Strand 2021). In psychiatry, eating pathology is typically approached as a reflection of something else—e.g., anxiety, impulsivity, or low self-esteem—where restrictive eating or binge eating become surrogate modes of expressing and managing affects that cannot be effectively dealt with in other ways. Food is seen merely as a medium, a stage upon which largely unrelated issues can be acted out. Eating (or not eating) becomes a metaphor (Skårderud 2007) or an idiom of distress (Kaiser and Weaver 2019)—a view that may hold intuitive merit but that also detracts from the centrality and materiality of food and eating in our everyday lives. A comparison can be made with research on substance use and addiction: admittedly, little is said about the nuances of various types of liquor in the biomedical literature on alcohol use disorder, but there is a substantial anthropological interest in topics such as drinking habits and culturally sanctioned alcohol intake (see, for example, Chapman 2020; Törrönen and Härkönen 2016; Castro et al. 2014; Bennett 1989). Ethnographic research on eating disorders, on the other hand, has typically focused on cultural aspects of body image or the lived experience of individuals with an eating disorder on hospital wards, online, etc., rather than on food and eating. A few notable exceptions specifically related to food, eating, and trauma have been discussed here (see also Abbots and Lavis 2013 and Abbots, Lavis, and Attala 2015 for further contributions that incorporate both symbolic and material aspects of food and eating disorders) but more qualitative and ethnographic research exploring these themes is certainly called for.

This study has been an attempt to address a research gap by bringing anthropological literature on food and trauma as related to identity, memory, and postmemory into dialogue with psychiatric research on eating disorders. Against that backdrop, however, a limitation of this article is that it has not engaged in depth with the research literature on trauma and *obesity*. Ethnographic and literary works are ripe with examples of how food is experienced as a comfort in the face of anxiety and stress. In the words of author Kiese Laymon: “[W]hen I was scared, I ran to cakes, because cakes felt safe, private, and celebratory. Cakes never fought back.” (Laymon 2018:123) This utterance should not be understood solely against a backdrop of abuse and relational trauma but also as embedded in a context of poverty, discrimination, and racism that contributes to day-to-day stress for large groups of the population (Carter 2007). It has been suggested that growing up in a socioeconomically deprived neighborhood may increase the risk of childhood obesity due to environmental factors such as street crime, traffic, and limited access to playgrounds and parks which may reduce walkability and opportunities for physical activity, although the patterns of association appears to be ambiguous (Cutts et al. 2009; Maroko et al. 2009; Showell et al. 2019). Neighborhoods with few supermarkets are sometimes referred to as *food deserts*—here, residents have to rely on corner stores and fast food establishments that typically provide energy-dense low-nutrient food items (Lopez 2007; Burdette and Hill 2008). Moreover, *food insecurity* is associated with both body dissatisfaction and eating pathology, especially binge eating behaviors. Tying in with the notion of an intertwining of scarcity and abundance, a “‘feast-or-famine’ cycle” has been suggested “in which food intake oscillates according to fluctuations in food availability, such that food intake decreases during periods of food scarcity and increases during periods of relative food abundance (e.g., after receiving a paycheck)” (Hazzard et al. 2020:74). A vicious cycle may ensue such that disadvantaged and vulnerable populations get blamed for making poor dietary choices when in reality they have few viable options; this ties in with what has been called “a complex ideology about blacks and their money that is compatible with the notion that black consumption is deviant behavior” (Austin 1994:228) as well as with the perpetual discussion of making more healthy options available for food stamp recipients. In parallel to the need for ethnographic research on trauma and eating disorders noted above, further research on the lived material realities of obese individuals, typically characterized by complex interactions between trauma, scarcity, and abundance, should become a priority.

This article has mostly dealt with psychological trauma in the wake of violent historical events such as colonialism, slavery, war, starvation, genocide, forced migration, and exile. In contrast, it has not provided much in-depth discussion about food in relation to other types of trauma of an interpersonal nature, such as childhood neglect, domestic violence, sexual abuse, or bullying. Although there has been research in the field of ethnology on topics such as food, cooking, and power relations within a family, this work has rarely touched upon family trauma and how it may be concretely manifested during mealtimes. As seen in the introduction section, there are examples from the anthropological literature of how family discord may be expressed through cooking (Nichter 1981; Stoller 1989) but the overall impression is that food in relation to interpersonal trauma is also an underdeveloped area in research.

In sum, this article has explored the nexus of food, memory, psychological trauma, and disordered eating in an effort to allow autobiographical narratives, works on food history, anthropology, and research in the field of eating disorders to collectively inform the understanding of the role of food and cooking in the wake of historical and personal trauma. This has included a large number of illustrations of how the dual nature of food and cooking—at once concrete and abstract, material and symbolic—offers an arena in which ambivalent memories of trauma can take on tangible form. Likewise, the two parallel and largely intertwined discourses of exoticism and hybridity have been evoked through a number of examples from food history. Specifically, the concept of postmemory may be useful in understanding how food and cooking can function both as a vehicle and a remedy for intergenerational trauma—here, the romanticized handing down of a cherished family recipe can, perhaps, be seen as a metaphor for intergenerational suffering as well as healing in a broader sense.

## Footnotes

1 Interestingly, anthropologist Mark Nichter’s original account of the concept of *idioms of distress* included actions such as serving a cold cup of coffee to a guest instead of the customary buttermilk and a spoon of the household’s finest mango pickle as one way of communicating marital discord among Havik Brahmins in South India (Nichter 1981).
